# Stereoselective Construction of β-, γ-, and δ-Lactam Rings via Enzymatic C–H Amidation

**DOI:** 10.21203/rs.3.rs-2429100/v1

**Published:** 2023-01-19

**Authors:** Satyajit Roy, David A. Vargas, Pengchen Ma, Arkajyoti Sengupta, Ledong Zhu, K. N. Houk, Rudi Fasan

**Affiliations:** a Department of Chemistry, University of Rochester, 120 Trustee Road, Rochester, New York, 14627, United States; b Current affiliation: Process Research and Development, Merck & Co., Inc., Rahway, NJ, 07065, USA.; c Department of Chemistry and Biochemistry, University of California, Los Angeles, California, 90095, United States; d School of Chemistry, Xi’an Key Laboratory of Sustainable Energy Materials Chemistry, MOE Key Laboratory for Nonequilibrium Synthesis and Modulation of Condensed Matter, Xi’an Jiaotong University, Xi’an, China; e Environment Research Institute, Shandong University, Qingdao 266237, PR China

## Abstract

Lactam rings are found in many biologically active natural products and pharmaceuticals, including important classes of antibiotics. Given their widespread presence in bioactive molecules, methods for the asymmetric synthesis of these molecules, in particular through the selective functionalization of ubiquitous yet unreactive aliphatic C–H bonds, are highly desirable. In this study, we report the development of a novel strategy for the asymmetric synthesis of 4-, 5-, and 6-membered lactams via an unprecedented hemoprotein-catalyzed intramolecular C-H amidation reaction with readily available dioxazolone reagents. Engineered myoglobin variants serve as excellent biocatalysts for this transformation producing an array of β-, γ-, and δ-lactam molecules in high yields, with high enantioselectivity, and on preparative scale. Mechanistic and computational studies elucidate the nature of the C–H amination and enantiodetermining steps in these reactions and provide insights into protein-mediated control of regioselectivity and stereoselectivity. Using this system, it was possible to accomplish the chemoenzymatic total synthesis of an alkaloid natural product and a drug molecule in much fewer steps (7–8 vs. 11–12) than previously possible, which showcases the power of this biosynthetic strategy toward enabling the preparation of complex bioactive molecules.

## Introduction

**Figure 1.**
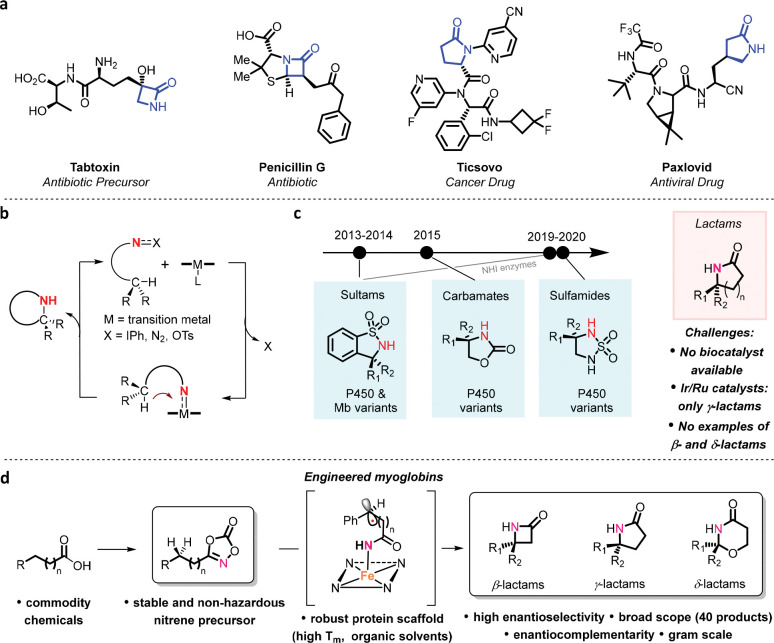
Asymmetric lactam synthesis via enzyme catalyzed C(*sp*^*3*^)-H amidation. **a)** Bioactive molecules containing β- and γ-lactam rings. **b)** General catalytic cycle for C-N bond formation via nitrene transfer **c)** Enzyme-catalyzed intramolecular C–H amination reactions. **d)** Biocatalytic construction β-, γ-, and δ-lactam rings via myoglobin-catalyzed intramolecular C–H amidation of dioxazolones (this work).

The selective functionalization of ubiquitous yet unreactive carbon-hydrogen (C–H) bonds via both chemical and enzymatic methods constitutes a powerful strategy for the diversification of organic molecules and enabling the devise of new disconnections and routes for the construction of complex molecules and natural products.^1–7^ Due to the large prevalence of amine-based functionalities in bioactive molecules and pharmaceuticals, a highly desirable and sought-after transformation in organic and medicinal chemistry is the selective amination of aliphatic C–H bonds.^8–10^ Notable advances in this field have led to the development of organometallic catalysts for catalyzing the insertion of nitrene species into C−H bonds, resulting in the formation of new carbon-nitrogen bonds (Figure 1b).^8–10^ These transformations are mediated by reactive metal-nitrenoid species generated upon reaction of the transition metal catalyst with nitrene precursor reagents such as iminoiodinane, azides, and hydrolamine derivatives.^8–10^ Using this strategy, a variety of cyclic amines, including oxazolidinones, sulfamates, sultams, and pyrrolidines have been accessible. Despite this progress, extension of this C–H amination strategy to the synthesis of cyclic amides (lactams), which are key structural motifs in many pharmaceuticals, agrochemicals, and other fine chemicals (Figure 1a),^11,12^ has represented a major challenge.^13^ The difficulty of this transformation can be attributed to the instability of the acyl nitrene intermediate that undergoes facile decomposition to isocyanates through a Curtius-type rearrangement, thereby outcompeting the desired C–H nitrene insertion process.^13^ Recently, Chang and coworkers has reported a breakthrough in this area through the development of an iridium-based system for enabling this transformation.^13^ This progress notwithstanding, asymmetric versions of this methodology are restricted to 5-membered rings (γ-lactams) and require the use of rare and toxic metals (i.e., Ir, Ru).^14,15^

Inspired by the chemistry of metalloporphyrins,^16^ our group and the Arnold group have recently demonstrated the ability of engineered hemoproteins to serve as biocatalysts for intramolecular^17–22^ and intermolecular^23–25^ C-H aminations via nitrene transfer (Figure 1c). Specifically, engineered cytochrome P450 enzymes have been shown catalyze the cyclization of sulfonyl azides, carbonazidates, and sulfonazidates^26^ to produce sultams, cyclic carbamates, and cyclic sulfamides, respectively.^17–22^ Furthermore, iridium-substituted P450s^27^ and non-heme Fe-dependent enzymes^28,29^ were also found to catalyze similar intramolecular C-H amination reactions. Despite this progress, the synthesis of lactam rings using biocatalytic nitrene transfer approaches has remained elusive, largely due to the aforementioned difficulty in controlling the reactivity of acyl nitrene intermediates while disfavoring other competing, unproductive reactions (e.g., nitrene reduction) known to affect these abiological enzyme-catalyzed reactions.^21^

Here, we report the development of a general biocatalytic methodology for the asymmetric synthesis of enantioenriched lactams via intramolecular C-H amidation of dioxazolones (Figure 1d), a safe and readily accessible class of nitrene donor reagents.^30^ This strategy provides an efficient and scalable approach to the selective construction of γ-lactam molecules with high enantioselectivity. Furthermore, the scope of this biocatalytic system could be extended to the construction of optically active β- and δ-lactam motifs with high enantiocontrol. The power of this methodology is further showcased through the implementation of concise chemoenzymatic routes for the total synthesis of an alkaloid natural product and a drug molecule. Comprehensive mechanistic studies elucidate the nature of the C–H amination step as well as the role of the protein scaffold in controlling the regio- and enantioselectivity of the reaction. Leveraging a direct C–H amination strategy, this methodology is mechanistically distinct yet complementary to recently reported biocatalytic approaches for γ-lactam synthesis that rely on radical-mediated cyclizations of alkene-containing substrates^31–33^ or carbene C–H insertion with artificial enzymes.^34^

## Results and Discussion

### Biocatalyst discovery.

Inspired by prior work of Chang and coworkers,^13,35^ we envisioned the possibility to execute an enzyme-catalyzed γ-C–H amidation reaction via nitrene transfer with dioxazolone reagent **1a** (Figure 1a–b). Particularly attractive features of dioxazolones as nitrene precursors include their facile synthesis from commodity carboxylic acids and their stability and safety compared to azide-based reagents previously used in biocatalytic nitrene transfer reactions.^17–22^ To identify an initial biocatalyst for this reaction, we tested various heme-containing enzymes and proteins, including wild-type myoglobin (Mb), cytochromes P450s, peroxidases, and cytochromes *c*, under anaerobic conditions (**Table S1**). While many of these reactions produced the acyclic amide **2b** as byproduct, none of these biocatalysts displayed any activity toward formation of the desired γ-lactam product (**Table S1**). Previous studies from our group demonstrated that mutations of the distal His64 residue in Mb (Figure 1c) can enhance its activity toward non-native carbene and nitrene transfer reactions.^36,37^ Promisingly, Mb(H64V) was found to react with **1a** to yield minute yet detectable amounts of the desired lactam **2a** (2% GC yield) with good levels of enantioselectivity (96% *ee*; Figure 1d).

**Figure 2.**
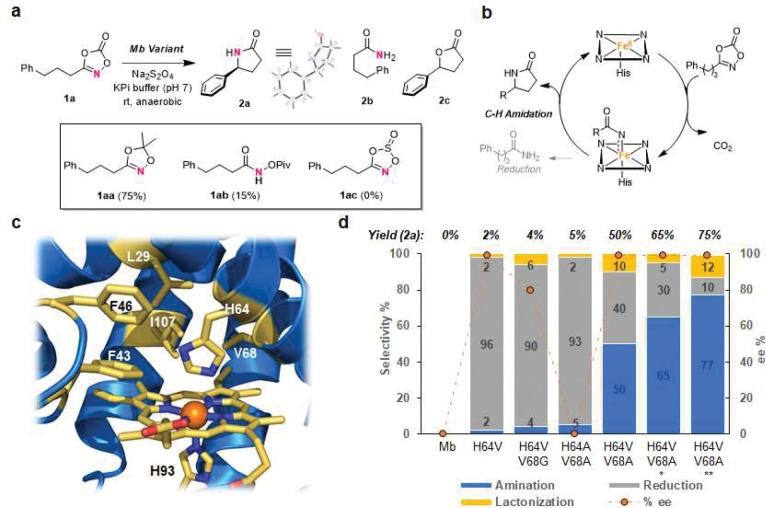
Biocatalytic intramolecular C(sp^3^)–H amidation with dioxazolones. (**a**) Reaction with 3-phenylpropyl-dioxazolone **1a**. *Box*: Structures of alternative nitrene precursors and corresponding yields for **2a** using Mb* under standard reaction conditions. (**b**) Envisioned hemoprotein-catalyzed nitrene transfer process for γ-lactam ring formation. (**c**) Crystal structure of wild-type Mb (pdb 1JW8) with residues near the Fe center highlighted in yellow. (**d**) Activity, chemoselectivity, and enantioselectivity (for **2a**) of engineered Mb variants in the reaction with **1a**. Reaction conditions: 20 μM protein, 10 mM **1a**, 10 mM Na_2_S_2_O_4_ in potassium phosphate buffer (50 mM, pH 7), 3 hours, room temperature, under anaerobic conditions. Yields and product distribution as determined by GC using calibration curves with isolated product. * With acetonitrile as co-solvent. ^**^ Using sodium borate buffer (pH 9) with 5% (v/v) acetonitrile (= standard reaction conditions or s.r.c).

Encouraged by these results, we extended the screening to a broader panel of engineered Mb variants (**Table S2**) containing mutations at the level of the distal His64 residue (Ala, Val, Gly) along with additional mutations within the active site of the hemoprotein (Figure 2c). From this screening, multiple Mb variants were found to exhibit increase C–H amidation activity compared to Mb(H64V) (10–50% yield; **Table S2**). Among them, Mb(H64V,V68A) (called Mb*), which was previously developed for stereoselective cyclopropanation,^36^ emerged as the best biocatalyst for this reaction, producing **2a** in 50% yield with excellent enantioselectivity (>99% *ee*) (Figure 2d). The configuration of the γ-lactam product was determined to be *S* by crystallography (Figures 2a and **S11**). In addition to the C–H amidation product, the Mb*-catalyzed reaction also produced a significant amount (40%) of the amide byproduct **2b** (Figure 2a), which likely arises from reduction and protonation of the nitrene intermediate (**Scheme S1**) as observed previously for C–H amination reactions with azide-based precursors.^18,22^ Unexpectedly, a minor product (10%) corresponding to the γ-lactone **2c** was also formed in the reaction (Figure 2a).

### Method optimization.

Compared to Mb*, Mb variants containing Ala and/or Gly mutations at the 64 or 68 positions showed significant decreases in activity and/or selectivity (Entries 3–6 vs. 7; **Table S2**) and a similar effect was observed upon introduction of additional mutations to Mb*, suggesting that the reaction is sensitive to subtle changes in the shape of the enzyme’s active site. Based on prior mechanistic studies on P450-mediated C–H amination^21^ and given the robustness of Mb biocatalysts to organic solvents,^38^ we hypothesize that the addition of an organic co-solvent could favor the desired C–H amidation reaction by disfavoring formation of the amide byproduct **2b**. Accordingly, screening of various organic co-solvents showed that acetonitrile (ACN) is beneficial toward increasing the yield of the C–H amidation product (50→65% yield), while reducing the undesired reduction reaction (30%) and without affecting enantioselectivity (Figures 2d and **S1**). Further optimization of the reaction conditions revealed that slightly alkaline conditions (pH 9) further increase the enzyme’s C–H amidation activity (75% yield of **2a**) at the expenses of byproduct **2b** (10%), while retaining excellent enantioselectivity (Figure 2d). These trends are consistent with our hypothesis that formation of the amide byproduct **2b** involves protonation of the nitrene intermediate, which should be disfavored under more alkaline conditions and in the presence of organic solvent. Under catalyst limiting conditions (0.07 mol%), Mb* was determined to catalyze the C–H amidation of **1a** in 58% yield and 2,175 TON with excellent enantioselectivity. In addition to dioxazolone **1a**, we also evaluated other nitrene precursors such as dioxazole **1aa**, acyl-protected hydroxylamine **1ab**, and dioxathiazole **1ac** (Figure 2a). Whereas **1ac** and **1ab** were inactive and less effective than **1a**, respectively, for the cyclization reaction (Figure 2c), dioxazole **1aa** produced the desired γ-lactam product in good yield (75% **2a**), thereby constituting another viable nitrene precursor for this biocatalytic C-H amidation strategy.

### Substrate scope.

**Figure 3.**
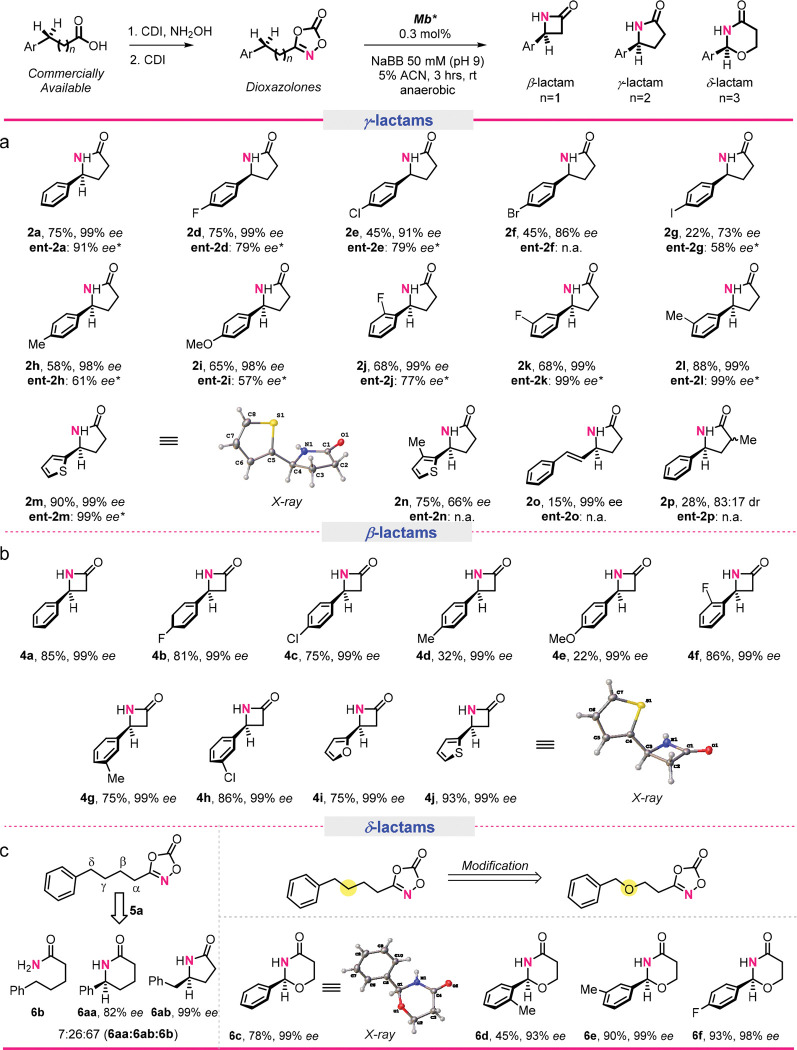
Substrate scope for the synthesis of γ-lactams **(a)**, β-lactams **(b)**, and δ-lactams **(c)** via Mb*-catalyzed asymmetric C-H amidation. * Using Mb(L29T,H64T,V68L) as the catalyst. n.a. = not active.

To assess the substrate scope of this methodology, Mb* was tested against an array of substituted dioxazolone substrates (**1d-p**). As summarized in Figure 3a, these experiments revealed a large tolerance of the enzyme toward substitutions at the *para* position of the aryl ring affording the desired γ-lactam products (**2d-i**) with high to excellent enantioselectivity (73–99% *ee*). While both electron-withdrawing and donating substitutions are accepted by the enzyme, increased activity was observed for substrates containing electron-donating substituents (**1h-i**). Substitutions at the *ortho* and *meta* positions were also well tolerated by the enzyme, producing the corresponding γ-lactam products (**2j-l**) in good yields (68–88%) and high enantiopurity (99% *ee*, Figure 3a). In addition to substituted phenyl groups, the enzyme is able to catalyze the cyclization of substrates containing heteroaryl groups such as thiophenyl group (**2m-n**) with good to high levels of activity (75–90% yields) and enantioselectivity (66–99% *ee*). Allylic C–H amidation was also possible as demonstrated by the successful preparation of **2o** in 99% *ee*. Lastly, enzymatic synthesis of **2p** in 28% yield and 83:17 diastereomeric ratio showed the tolerance of the enzyme also to substitutions in α to the dioxazolone core.

### Enantiodivergent biocatalyst.

Enantiodivergent biocatalysts are highly desirable yet often hard to develop.^39^ Notably, screening of the initial Mb active-site mutant library revealed a variant, Mb(L29T,H64V,V68L), that catalyzes the cyclization of **1a** with inverted enantioselectivity compared to Mb*, producing the *R*-configured γ-lactam product **ent-2a** in 65% *ee*, albeit in modest yield (15%; **Table S2**). To improve the performance of this biocatalyst, Mb(L29T,H64V,V68L) was subjected to active-site mutagenesis, ultimately leading to Mb(L29T,H64T,V68L), which produces **ent-2a** with both an improved enantioselectivity of 91% *ee* and two-fold higher activity compared to the parent enzyme. To explore the substrate promiscuity of this enantiocomplementary biocatalyst, Mb(L29T,H64T,V68L) was tested against the panel of dioxazolones **1d-p**. Albeit in more moderate yields compared to the Mb* reactions, the majority of these substrates (10/14) could be converted to the *R*-configured γ-lactam products with good to high enantioselectivity (57–99% *ee*; Figure 3a). Of note, **ent-2k**, **ent-2l**, and the thiophenyl containing substrate **ent-2m** were all obtained in 99% enantiomeric excess. Altogether, these results highlighted the broad substrate scope and predictable enantiocomplementarity of the two Mb-based biocatalysts for γ-lactam ring formation.

### Synthesis of β-lactams and δ-lactams.

Next, we targeted the synthesis of β-lactams which are highly desirable building blocks for medicinal chemistry as well as key pharmacophores in β-lactam antibiotics.^12^ Notably, β-lactam formation via intramolecular nitrene transfer has not been reported to date. Upon challenging Mb* with substrate **3a**, the desired β-lactam **4a** was obtained in high yield (85%) and excellent enantioselectivity (99% *ee*) (Figure 3b). Mirroring the *S*-enantioselectivity in γ-lactam formation, the enzyme maintains *S*-enantiopreference for the formation of **4a**, as determined by X-ray crystallography (Figure 3c). These findings prompted us to further explore the substrate scope of this reaction (**3b-j**; Figure 3b). Remarkably, variously substituted substrates could be converted into the desired β-lactam products with excellent enantioselectivity (99% *ee*; Figure 3b) and up to 93% yield. Unlike the γ-lactams, substrates bearing electron-withdrawing groups on the aryl ring were cyclized more efficiently than those containing electron-donating groups (e.g., 32% yield for **4d** vs. 75% for **4c**) and *para* substitutions were better tolerated than *meta* substitutions (e.g., 75% yield for **4g** vs 32% for **4d**). These differences likely arise from the differential role of electronic and steric constraints in the 4- vs. 5-membered ring formation. Furanyl- and thiophenyl-containing substrates **3i** and **3j**, respectively, were also cyclized very efficiently (75% and 93% yields, respectively) and with high enantiocontrol (99% *ee*).

To explore the reactivity of the Mb biocatalyst toward synthesis of δ-lactams, a reaction was carried out using substrate **5a**, which resulted in a mixture of δ-lactam (**6aa**) and γ-lactam (**6ab**) in a 1:3.7 ratio, in addition to the amide **6b** as the major product (7:26:67 ratio for **6aa**:**6ab**:**6b**; Figure 3c). These results revealed the enzyme’s preference for amidation of the homobenzylic γ-C–H bond (to give **6ab**) over the benzylic δ-C–H bond (to give **6aa**) despite the higher bond dissociation energy (BDE) of the latter (~95 vs. 90 kcal/mol) (Figure 3c). These findings inspired us to substitute the γ-C–H bond with an O atom to favor δ-lactam formation. Gratifyingly, the Mb* reaction with **5c** produced the desired δ-lactam **6c** with significantly improved efficiency (78% yield) as well as excellent enantioselectivity (99% *ee*; Figure 3c). This biocatalytic reaction was also found to be tolerant toward substitution on the aryl ring, as demonstrated by the synthesis of δ-lactams **5d-f** in 45–93% yields and high enantiomeric excess (90–99% *ee*). Taken together, these results reveal a remarkable generality of the Mb* catalyst toward enabling the stereoselective synthesis of lactams of varying sizes and with different substitutions. Noteworthy is also the consistent and predictable *S* stereoselectivity of the Mb*-catalyzed C–H amidation reaction not only across the different substrates but also across the β-, γ-, and δ-lactam rings, which adds to the synthetic utility of this biocatalytic system.

### Mechanistic Studies.

**Figure 4.**
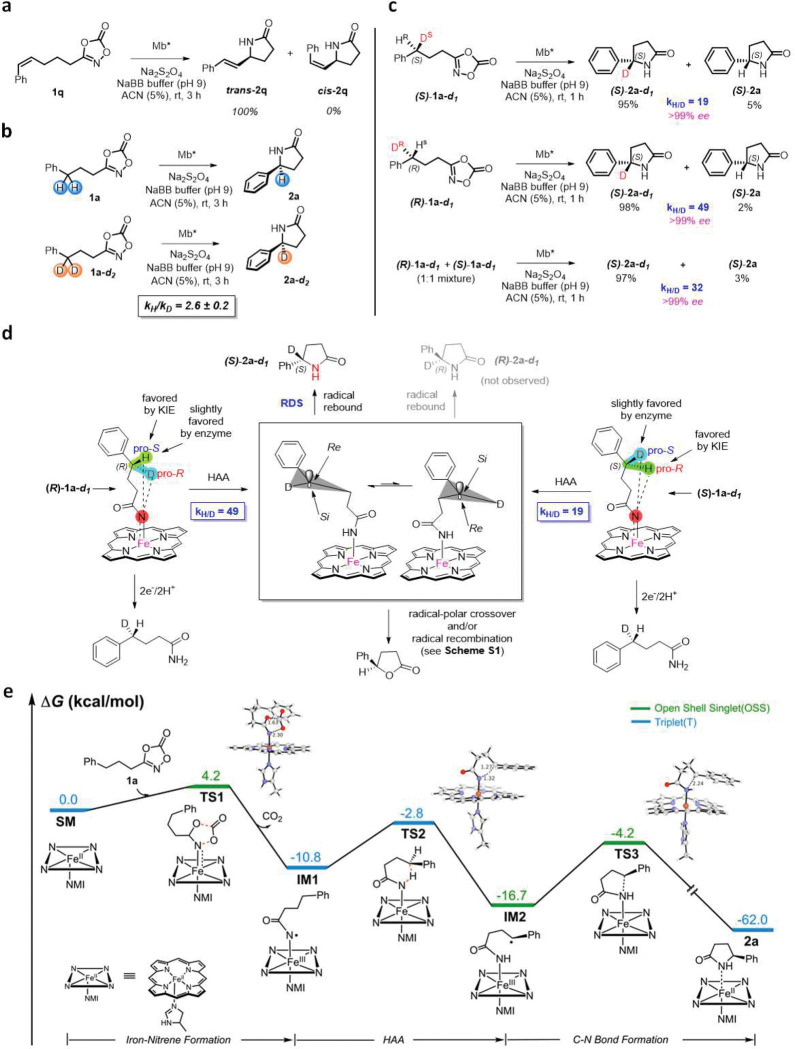
Mechanistic studies. **a)** Mb-catalyzed cyclization of substrate **(*Z*)-1q** shows complete isomerization to the *E* isomer product ***trans*-2p** (see **Figure S2** for details). **b)** Non-competitive intermolecular KIE experiments as determined via comparison of product formation rates from parallel reactions with protiated and deuterated substrate. See **Figure S3** for details. **c)** H/D competition experiments with enantiopure isotopomeric dioxazolidinones. K_H/D_ values were determined by NMR (see **Figure S4** for details). **d)** Mechanistic model for Mb*-controlled asymmetric induction. HAA = H atom abstraction. **e)** Gibbs free energy diagram for the Mb-catalyzed γ-lactam formation. ΔG values were calculated at the uB3LYP-D3BJ/def2tzvp(SMD, solvent=water)//uB3LYP-D3BJ/6–31G(d)+SDD(Fe) level using a truncated computational model of the enzyme (see Supporting Information for details). See **Figure S5** for additional spin states. NMI=5-methyl-imidazole. Distances in Å.

Studies were then performed to gain insights into the mechanism of this enzyme-catalyzed reaction. To probe the nature of the C–H amidation step, the Mb*-catalyzed reaction was carried out in the presence of the *Z*-configured dioxazolone **1q**, which resulted in the formation of the lactam product **2q** in the *E* configuration (Figure 4a). This result rules out a concerted C–H nitrene insertion process and is consistent with a stepwise hydrogen atom abstraction (HAA)/radical rebound mechanism proceeding via an allylic radical that undergoes *Z*→*E* isomerization to yield ***trans***-**2q** prior to radical recombination. Of note, complete isomerization of the double bond in the cyclization product (no *cis*-**2q** was observed) shows that the radical intermediate is relatively long-lived.

To further investigate the kinetic role of the C–H cleavage step, non-competitive intermolecular H/D competition experiments were carried out using substrate **1a** and **1a-*d***_***1***_ in parallel reactions (Figure 4b). These experiments yielded a kinetic isotope effect (KIE) value of 2.6 ± 0.2 (Figure 4b and **S3**), which is lower than that determined for C−H amination reactions with azide-based substrates catalyzed by engineered P450s (*k*_H_/*k*_D_: 3.4–5.3)^20,40^, but higher than that determined for P450-catalyzed cyclization of sulfonylazides (*k*_H_/*k*_D_: 0.9), where the azide activation was established to be rate-determining.^21^ Overall, these results indicated that the C–H cleavage step in the present system is only partially rate determining, with other steps contributing to control the overall rate of the reaction.

To gain insight into the enantioselectivity determining step(s) in this reaction, isotopomeric dioxazolidinones (*S*)-**1a-*d***_***1***_ and (*R*)-**1a-*d***_***1***_ were synthesized in enantiopure form (**SI Schemes SX-X**) and subjected to Mb*-catalyzed C–H amidation. Interestingly, both reactions led to the accumulation of deuterated lactam (*S*)-**2a-*d***_***1***_ as the largely predominant product over the protiated counterpart (*S*)-**2a** (95:5 and 98:2 ratio from (*S*)-**1a-*d***_***1***_ and (*R*)-**1a-*d***_***1***_, respectively) as determined by NMR (Figure 4c and **S4**). In addition, both reactions proceed with high enantioselectivity (99% *ee*). From these results, it can be derived that (a) H abstraction is strongly favored over D abstraction regardless of the configuration of the C–H amination site, and (b) protein-mediated enantioinduction in the C–N bond forming process must occur at the level of the radical rebound step (Figure 4d). Indeed, formation of (*S*)-**2a-*d***_***1***_ as the major product from either (*S*)-**1a-*d***_***1***_ or (*R*)-**1a-*d***_***1***_, along with the preserved high *% ee* in both cases, imply that, after HAA, the pro-*S* and pro-*R* conformations of the radical intermediate can undergo rapid interconversion, with the enzyme active site enforcing radical rebound through the pro-*S* intermediate (*Si* face attack) (Figure 4c).

The k_H/D_ values derived from the reactions with the isotopomeric substrates (Figure 4c) result from a combination of the KIE (*k*_H_/*k*_D_) and enzyme’s enantiopreference in the HAA step (i.e., *k*_S_/*k*_R_). The larger k_H/D_ measured for (*R*)-**1a-*d***_***1***_ vs. (*S*)-**1a-*d***_***1***_ (49 vs. 19) indicates a higher chirality-match for H abstraction in the former substrate vs. the latter, i.e., abstraction of H^pro-*S*^ in (*R*)-**1a-*d***_***1***_ is favored by both KIE and the enzyme, whereas abstraction of H^pro-*R*^ in (*S*)-**1a-*d***_***1***_ is favored by KIE but disfavored by the enzyme (Figure 4d). However, the mere 2.5-fold difference between the k_H/D_ values (in constrast to the 50- to 220-fold difference determined with other systems^40,41^) suggests that the HAA step is barely stereoselective and thus that asymmetric induction is largely controlled by the enzyme during the radical recombination step. As such, this system shows a distinct enantioinduction mechanism compared to that described for asymmetric C–H aminations of sulfonyl azide substrates catalyzed by Co-porphyrins^41^ and P411 catalysts^22,40^.

### Computational analysis (DFT) of the reaction mechanism.

Density functional theory (DFT) calculations were performed to explore the formation of the γ-lactam product **2a** from dioxazolone **1a**. The quintet (high spin), triplet (intermediate spin), closed-shell singlet (low spin) and open-shell singlet (low spin) spin states of each intermediate and transition state structure were considered in the calculations. Figure 4e reports energies of the lowest energy spin-state for different intermediates and transition-states, with energies of other spin states (**Figure S5**) and computational details in the Supporting Information. DFT calculations describe the iron-nitrenoid active species in this system as a triplet ground state, in analogy with what found for other iron-nitrenoid species.^22,25^ Calculations indicate that formation of iron-nitrenoid complex **IM1** through activation of dioxazolone substrate, and concomitant loss of CO_2_, has an energy barrier of only 4.2 kcal/mol and involves an open shell singlet (OSS) spin state. After spin crossing, the hydrogen atom abstraction (HAA) step is favored in the triplet state, giving a carbon radical intermediate **IM2**, with an 8.0 kcal/mol energy barrier. The following step, involving a radical rebound to give the final γ-lactam product, was found to be most favorable in the open shell singlet state with an energy barrier of 12.5 kcal/mol. The **IM2** has a heavy spin contamination (S^2^=1.10), indicating that the OSS is not a pure singlet spin state, but 50:50 singlet:triplet, often observed in calculations on diradicals. Based on the energy profile, the C-N bond forming step is predicted to be rate determining, exhibiting a 4.5 kcal/mol higher energy barrier than the C–H bond cleavage event. These findings are in excellent agreement with the results from the mechanistic experiments (Figure 4) and explain the long-lived nature of the radical intermediate, as suggested by the complete double bond isomerization observed with ***trans***-**2q** (Figure 4a).

This mechanistic model provides a framework also for defining a plausible mechanism for the formation of the unexpected γ-lactone product **3c** from **1** (Figure 2a), a reaction that finds no precedents in catalytic nitrene transfer reactions. After the HAA step, single electron transfer from the C-centered radical to the heme (or protein matrix) can generate a benzylic carbocation which can then react with the amide group (via the carbonyl group) to form a dihydrofuranimine ring. Hydrolysis of the latter produces **3c** (**Scheme S1**). In addition to this radical-polar crossover mechanism, a radical pathway can be envisioned that proceeds via the same dihydrofuranimine intermediate produced via reaction of the benzylic radical with the amidyl group (**Scheme S1**). In either case, a slow C-N bond forming radical rebound step as revealed by our mechanistic and DFT studies is expected to enable this competing side reaction to occur under suboptimal reaction conditions.

### Enzyme-controlled regio- and enantioselectivity.

**Figure 5.**
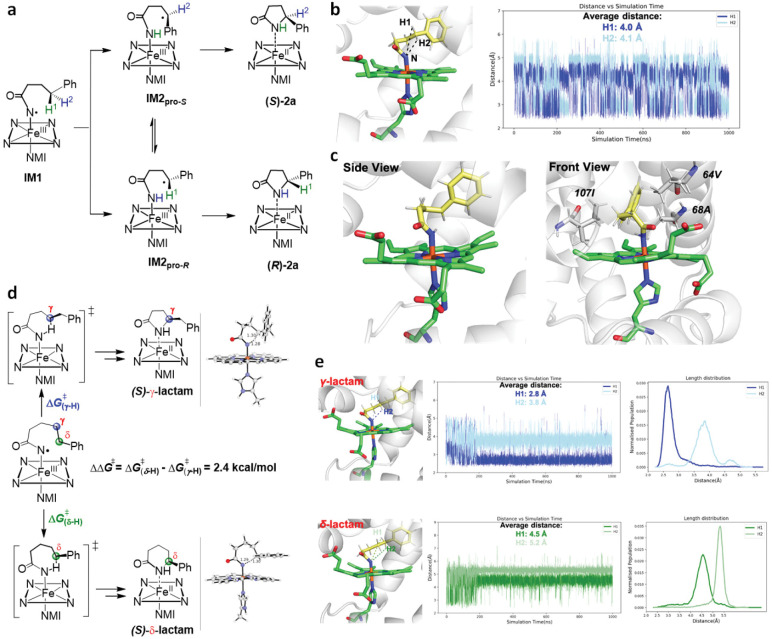
Computational analysis of Mb*-induced enantio- and regiocontrol in C–H amidation. **a**. H atom abstraction step in the C–H amidation of **1a** to give the corresponding γ-lactam product. **b**. Representative snapshot of IM1 intermediate in Mb* active site and plot of the nitrene N∙∙∙H^1^ and nitrene N∙∙∙H^2^ distances over the entire 1000 ns MD simulations. See **Figure S6** for additional replicas. **c**. Most populated conformation of the benzylic radical intermediate IM2 in Mb* heme pocket over the entire 1000 ns simulation. Key residues implicated in stabilizing IM2 conformation leading to the *S*-configured product **2a** after radical rebound are highlighted. **d**. DFT calculated energy barrier difference for γ-H vs. δ-H atom abstraction by nitrene intermediate IM1 derived from substrate **5a**. Structures of the transition states are shown with key distances given in Å. **e**. Representative snapshots of **5a**-derived IM1 intermediate in Mb* active site and distance plots and Boltzmann distribution of the nitrene N∙∙∙γ-H atoms and nitrene N∙∙∙δ-H atoms distances over the entire 1000 ns MD simulation. See **Figure S9** for additional replicas.

To investigate the role of the enzyme in controlling the enantioselectivity of the reaction, we explored the heme-bound iron-nitrenoid intermediate **IM1** docked in the active site of Mb* using the available crystal structure of this protein.^42^ As shown in Figure 5a, the N atom of **IM1** can abstract either the H^1^ or H^2^, leading to the *S* and *R* lactam product, respectively, under fast rebound conditions. We measured the evolution of distances between the N atom and the H^1^ and H^2^ atoms during 1000 ns MD simulations (Figure 5b). These studies show that the average distance from N atom to H^1^ and H^2^ are 3.96–4.01 Å and 4.06–4.10 Å, respectively, suggesting little to no preference for abstraction of H^1^ vs. H^2^ (leading to the *S* vs. *R* product) by the nitrene intermediate.

Next, the carbon radical intermediate **IM2** was docked in Mb* active site in both a conformation that could lead to the *S* lactam product (**IM2**_**pro(S)**_) and a conformation that could lead to the *R* lactam product (**IM2**_**pro(R)**_), as shown in **Figure S7**. After 1000 ns MD simulation, both complexes converged in a conformation of the intermediate that favors radical rebound through the *Si* face of the benzylic radical species (Figure 5c) leading to the (*S)*-γ-lactam **2a**. Inspection of this structure indicates that the active site residues Ile107, Val64 and Ala68 contribute to orient the heme-bound intermediate in the pro-(*S*) conformation. Furthermore, DTF calculations show that **TS3** for formation of the *R*-configured product is disfavored by 3.1 kcal/mol versus that leading to the *S* product (**Figure S8**). This ΔΔG^‡^ is consistent with the high *S*-enantioselectivity induced by the enzyme (>99:1 e.r.) in the intramolecular C–H amidation reaction. The models for both the HAA and radical rebound step show the aryl ring projecting into the core of the protein between Ile107 and Ala68 (Figure 5b–c). While this arrangement can accommodate substitution at different positions of the aryl ring, it also shows potential steric constraints as the size of the *para* substituent increases, providing a plausible rationale for the structure-activity trends observed experimentally (i.e., –F (**2d**) > -Cl/Br (**2e-f**) > -I (**2g**); Figure 4a).

To understand the regioselectivity of the enzyme, we studied the nitrene intermediate **IM1** generated from substrate **5a** (Figure 5d). In this case, the nitrene intermediate can abstract either the γ- or δ-H atom, leading to the 5- and 6-membered lactam product, respectively. DFT calculations show that the energy barrier for hydrogen abstraction leading to the δ-lactam is 2.4 kcal/mol higher than for the formation of γ-lactam (Figure 5d). We also studied the proximity of the γ and δ H atoms to the nitrene N atom via 1000 ns MD simulation. The γ-H…N average distance is 2.8–3.9 Å, whereas for δ-H…N distance is significantly larger, namely 4.3–5.2 Å (Figures 5e and **S10)**. Thus, both the lower energy barrier for H_γ_ abstraction and a closer nitrene N…H_γ_ atom distance contribute to favor formation of the γ-lactam product, which can explain the regioselectivity of the Mb*-catalyzed C–H amidation of **5a** observed experimentally (Figure 4c).

### Chemoenzymatic total synthesis of bioactive molecules.

**Figure 6.**
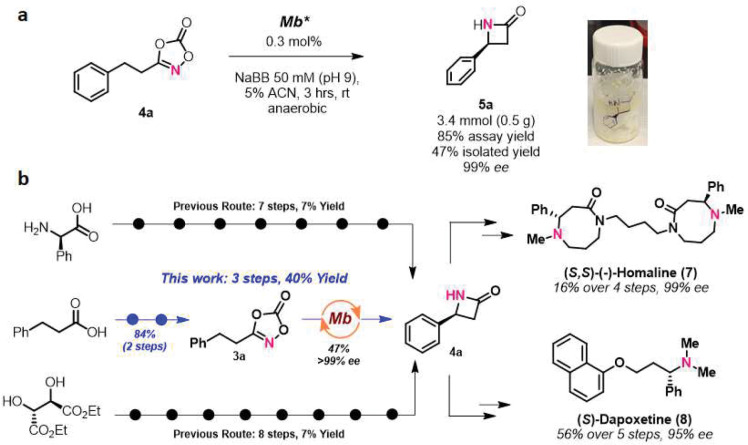
Chemoenzymatic total synthesis of natural producyt alkaloid (*S,S*)-(−)-Homaline (7) and synthetic drug (*S*)-Dapoxetine (8).

The present strategy was then applied for the chemoenzymatic syntheses of bioactive alkaloid (*S,S*)-(−)-Homaline (**7**) and FDA-approved drug (*S*)-Dapoxetine (**8**) (Figure 6). Specifically, we envisioned that the key β-lactam intermediate **4a**, previously accessible only in low yields and after lengthy routes (7–8 steps, 7% overall yield^43,44^; Figure 6b), could be produced in a more efficient and step-economical manner by enzymatic means using the present method. Accordingly, enantiopure β-lactam **4a** (>99% *ee*) was produced from **3a** and isolated on a preparative scale (0.5 g) from a scaled up reaction with Mb* (Figure 6a). From **4a**, (*S,S*)-(−)-Homaline (**7**) and (*S*)-Dapoxetine (**8**) could be synthesized in only four and five steps, respectively, using known routes (Figure 6b; see **Scheme S2** for further details). Overall, asymmetric Mb-catalyzed C–H amidation enabled the chemoenzymatic synthesis of the alkaloid natural product and drug molecule in a total of 7 and 8 steps, respectively, compared to 11 and 12 steps required in previous total synthesis strategies. In addition, with the present approach, the key enantiopure β-lactam intermediate **4a** was readily obtained from the achiral, commodity chemical hydrocinnamic acid as opposed to more expensive optically active precursors required in the previously reported routes (Figure 6b). These results further showcase the synthetic utility and scalability of the present methodology for the synthesis of biologically active molecules.

## Conclusions.

In summary, we have developed a first biocatalytic strategy for the asymmetric construction of lactam molecules via nitrene transfer. Starting from readily accessible dioxazolones, this strategy could be leveraged to afford a broad range of β−, γ-, and δ-lactam scaffolds in good yields and high enantiomeric excess using a single Mb-based biocatalyst. In addition, we demonstrated the possibility to obtain enantiopodes of the γ-lactam products using an alternate engineered Mb variant with enantiodivergent selectivity. Our mechanistic investigations revealed that these reactions proceed via a HAA/radical rebound pathway, with the enzyme binding site controlling the stereo- and regioselectivity (β-, γ-, δ-C-H amidation) of the process. Furthermore, while the HAA step is generally assumed to be enantiodetermining in enzymatic C–H aminations^45,46^, our studies show that protein-induced enantioselectivity in the present system is largely controlled at the level of the radical rebound step. The power of the present methodology was further showcased by the concise chemoenzymatic total synthesis of an alkaloid natural product and a drug molecule in about half of the steps required previously, while offering higher overall yields and starting from a commodity chemical instead of optically active precursors. This work expands the available biocatalytic toolbox for the asymmetric synthesis of amine-containing molecules and paves the way to the development of other asymmetric enzyme-catalyzed nitrene transfer reactions involving dioxazolones as nitrene precursors.

## Data Availability

Data that support the findings of this study are included in this published article (and its supplementary information files). Additional datasets generated during and/or analysed during the current study are available from the corresponding author on reasonable request. Crystallographic data for small molecules have been deposited in the Cambridge Crystallographic Data Centre (CCDC) as described in the supplementary information files.
